# Traditional Chinese medicine on treating splenomegaly due to portal hypertension in cirrhosis: A protocol for systematic review and meta-analysis

**DOI:** 10.1097/MD.0000000000024081

**Published:** 2021-01-08

**Authors:** Zhaodi Wang, Zhaoxing Chen, Zhipeng Fan, Yong Jiang

**Affiliations:** Chengdu University of Traditional Chinese Medicine, School of Basic Medical Sciences.

**Keywords:** hepatocirrhosis with splenomegaly, protocol, systematic review, traditional Chinese medicine

## Abstract

**Background:**

Liver cirrhosis is a common clinical chronic progressive disease. Due to the obstruction of blood flow after cirrhosis, it leads to long-term congestion of splenic sinus, hyperplasia of fibrous tissue and proliferation of splenic myeloid cells, resulting in hepatocirrhosis and splenomegaly. At present, western medicine still uses splenectomy and interventional therapy are the main treatment, but the adverse reactions are more and the curative effect is not good. Many clinical trials have proved that Traditional Chinese medicine has a great therapeutic effect on Hepatocirrhosis with splenomegaly, which can effectively delay the development of the disease and improve the survival rate of patients. This systematic review aims to evaluate the efficacy and safety of Traditional Chinese medicine in the treatment of hepatocirrhosis with splenomegaly.

**Methods:**

The databases of Pubmed, CENTRAL (The Cochrane Central Register of Controlled Trials), China National Knowledge Infrastructure (CNKI), Wanfang Data Knowledge Service Platform (WANFANG Data), Weipu Information Chinese Periodical Service Platform (VIP), and China Biomedical Literature Service System (SinoMed) will be searched online to collect randomized controlled trials related to the treatment of hepatocirrhosis with splenomegaly with Traditional Chinese medicine The time is limited from the construction of the library to November 2020. We will use the criteria provided by Cochrane 5.1.0 for quality assessment and risk assessment of the included studies, and use the Revman 5.3 and Stata 13.0 software so as to systematically review the effectiveness of Traditional Chinese medicine for hepatocirrhosis with splenomegaly.

**Ethics and dissemination:**

This systematic review will evaluate the efficacy and safety of Traditional Chinese medicine for hepatocirrhosis with splenomegaly. Because all data used in this systematic review and meta-analysis have been published, this review does not require ethical approval. In addition, all data will be analyzed anonymously during the review process.

**Results:**

In this study, we will evaluate the efficacy of Traditional Chinese medicine in the treatment of cirrhosis with splenomegaly.

**Conclusion:**

The conclusion of this study will be evidence to ensure the efficacy of Traditional Chinese medicine© in the treatment of cirrhosis with splenomegaly and provide guidance for its treatment.

**Trial registration number:**

INPLASY2020110121.

## 1 Introduction

Liver cirrhosis is a common clinical chronic progressive disease, which is caused by one or more pathological factors acting on the body for a long time or repeatedly.^[1,2]^ There are extensive hepatocyte necrosis, nodular regeneration of residual hepatocytes, connective tissue hyperplasia and fibrous septum formation, which lead to the destruction of hepatic lobular structure and the formation of pseudolobule.^[3]^ In 1900, Gilbert and Villarct et al of France measured that the ascites pressure of patients with liver cirrhosis was as high as 2.94-3.92 kPa (300–400 mmh2o), and the term portal hypertension was first proposed.^[4]^ Portal hypertension often leads to splenomegaly, The vast majority of splenomegaly will be accompanied by hypersplenism (referred to as hypersplenism).^[5]^ The liver gradually deforms and hardens and develops into cirrhosis. The disorder of liver blood circulation leads to portal hypertension and splenomegaly.

Liver cirrhosis can be divided into compensatory and decompensated stages. The early symptoms are not prominent, and the later stage is mainly manifested as the right flank pain and discomfort, loss of appetite, fatigue, abdominal distension, diarrhea, weight loss and other clinical symptoms.^[1]^ With the progress of the disease, patients will gradually appear a series of complications, such as splenomegaly, ascites, upper digestive tract hemorrhage, hepatic encephalopathy, hepatorenal syndrome and other complications, and finally due to multiple organ failure The prognosis is very poor.^[6]^ At present, many western medicine surgical treatment, such as splenectomy or partial splenic embolization, can improve hypersplenism and peripheral blood cell reduction to a certain extent, but it is still helpless for liver cirrhosis. And modern research found that western medicine surgery has more adverse reactions.^[7]^ As a result, people gradually regard it as an incurable disease. On the contrary, the effect of Traditional Chinese medicine on the disease is quite good, through the combination of theory, method and prescription, not only can improve hypersplenism, reduce portal pressure, but also can improve liver function, with satisfactory results.^[8–10]^

TCM is widely used clinically to treat hepatocirrhosis with splenomegaly. This disease is equivalent to “distension”, “jaundice” and “accumulation” in Traditional Chinese medicine.^[11]^ Different doctors have different understandings of its etiology and pathogenesis. In short, TCM believes that the cause of the disease lies in the internal invasion of the liver by external pathogens such as mood, diet, labor desire and epidemic toxin, resulting in liver laxation and dereliction of duty, stagnation of liver qi and spleen, stagnation of qi, blood, dampness and blood stasis. The general pathogenesis is deficiency of vital energy and blood stasis.^[12]^ Traditional Chinese medicine is mainly used for soothing the liver and strengthening the spleen, promoting qi and activating blood circulation, softening the hard and dispersing nodules, such as ginseng, astragalus, Poria cocos, angelica, Salvia miltiorrhiza, radix paeoniae rubra, peach kernel, Aucklandia, Fructus aurantii, bupleurum, turmeric, roasted armour, Sparganium, rhizoma curcumae, Scutellaria barbata, etc.^[13]^ Pharmacological studies confirmed that Traditional Chinese medicine mainly through strengthening the immune system, protecting liver cells, promoting liver cell regeneration, improving liver and spleen circulation, reducing portal hypertension, reducing spleen, inhibiting fiber proliferation and so on.^[14]^

## 2 Methods

### 2.1 Study registration

This systematic review protocol has been registered on INPLASY as INPLASY2020110121 (DOI: 10.37766/inplasy2020.11.0121). All of the data for the article have been published online, so this protocol does not require ethical approval. The protocol will be strictly enforced according the Guide to the contents of a Cochrane protocol and review (Part1.Chapter4 of Cochrane Handbook for systematic review of interventions Version 5.1.0).^[15]^

### 2.2 Inclusion criteria for study selection

#### 2.2.1 Types of studies

We will search all the studies that TCM is used as the main intervention for hepatocirrhosis with splenomegaly, Non-RCTs quasi-RCTs, series of case reports, and cross research will be excluded. Full article not available will be excluded. No language restrictions.

#### 2.2.2 Types of participants

All the patients who have been diagnosed with hepatocirrhosis with splenomegaly will be included. There are no restrictions on age, gender, regional, national, belief, ethnic, sources, and courses of disease.

#### 2.2.3 Types of interventions

There is no requirement for the intervention course, the specific contents of the control group and the experimental group are as follows.

##### 2.2.3.1 Control intervention

The control group received conventional western medicine or surgical treatment, including nucleotide drugs, intravenous drip of albumin, or oral amino acid, splenectomy. Specific drugs, doses and methods are not limited. If the control group was treated with Traditional Chinese medicine, the study was excluded.

##### 2.2.3.2 Experimental interventional

The experimental group is treated with TCM on the basis of conventional western medicine treatment in the control group. The use of TCM is limited to prescription and Chinese patent medicines. Prescription drugs require a clear dose, but there are no restrictions on the composition, dosage form and dosage. For the dosage, such as decoctions, granules, pills, powders, etc. Other types of TCM treatments such as TCM injections, acupuncture, moxibustion, massage, cupping, and others will be excluded.

### 2.3 Outcome measures

#### 2.3.1 Primary outcomes

The primary outcomes are total effective rate (total effective rate = significant efficiency + effective rate), liver function (ALT, AST, ALB, TBIL) and liver and spleen color to exceed.

#### 2.3.2 Secondary results

The secondary evaluation criteria were as follows: liver and spleen B ultrasound; serum fibrosis indicators: HA, PCIII, IV-C, LN. More importantly, adverse reactions during the trial will also be recorded.

### 2.4 Exclusion criteria for study selection

1. The studies without primary outcomes;
2. the studies that cannot be obtained in full text or no data can be extracted;
3. the studies with obvious errors in the data;
4. the one with the higher cases will be selected, while the studies have the same data source as the cases;
5. for duplicate publications or similar studies, just select one of them.

### 2.5 Searching strategy

#### 2.5.1 Electronic searches

The databases of PubMed, CENTRAL (The Cochrane Central Register of Controlled Trials), Excerpt Medical Database (Embase), China National Knowledge Infrastructure (CNKI), Wanfang Data Knowledge Service Platform (WANFANG Data), Weipu Information Chinese Periodical Service Platform (VIP), and China Biomedical Literature Service System (SinoMed) will be searched online. The search time is set from the establishment of the search database to November 2020. According to the standards of the Cochrane Collaboration workbook of the International Evidence-Based Medicine Center, the search terms include “Chinese medicine,” “Traditional Chinese medicine,” “proprietary Chinese medicine,” “Chinese herbal medicine,” “splenomegaly,” and “Liver Cirrhosis.” The complete PubMed search strategy is summarized in Table 1.

**Table 1:** Search strategy used in PubMed database.

Number	Search terms
1	Search (Traditional Chinese Medicine) OR ((((((((((((Chung I Hsueh) OR Traditional Medicine, Chinese) OR Zhong Yi Xue) OR Chinese Traditional Medicine) OR Chinese Medicine, Traditional) OR Traditional Tongue Diagnosis) OR Tongue Diagnoses, Traditional) OR Tongue Diagnosis, Traditional) OR Traditional Tongue Diagnoses) OR Traditional Tongue Assessment) OR Tongue Assessment, Traditional) OR Traditional Tongue Assessments)
2	Search (cirrhosis) OR (((((Hepatic Cirrhosis) OR Cirrhosis, Hepatic) OR Cirrhosis, Liver) OR Fibrosis, Liver) OR Liver Fibrosis)
3	Search (Splenomegaly) OR ((Enlarged Spleen) OR Spleen, Enlarged)
4	Search Randomized controlled trial
5	1 and 2 and 3 and 4

#### 2.5.2 Searching other resources

The manual is search mainly for ongoing experiments, and grey literature. We will look for abstracts and conference papers related to Traditional Chinese medicine and hepatocirrhosis with splenomegaly. Ongoing experiments which have been registered on World Health Organization International Clinical Trials Registry Platform (ICTRP) or Chinese Clinical Trial Registry. Try to contact the researchers to inquire about the progress of the trial and provide the latest test data. As for grey literature, we will retrieve on the following websites: Grey Net International, SIGLE (The System for Information on Grey Literature in Europe), Open Gery, Gery Literature Report. The secondary literature search will be performed on the included references to reduce the omission factor.

### 2.6 Data collection and analysis

#### 2.6.1 Study description

**Figure 1:**
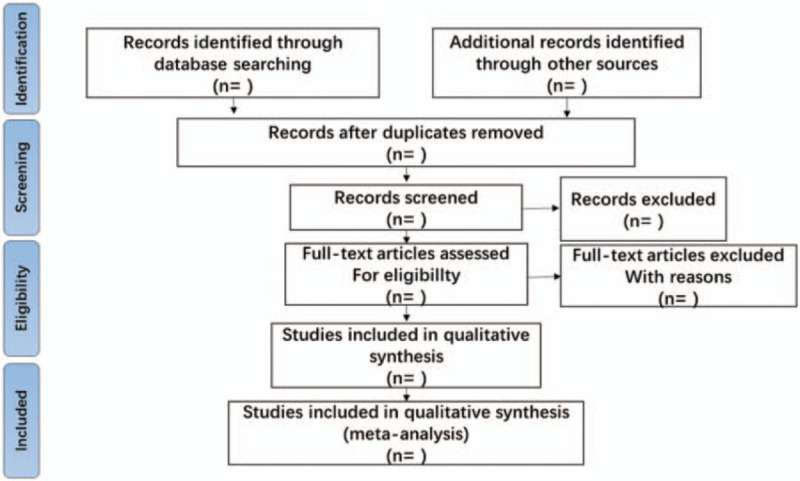
The PRISMA flow chart.

1. The initial screening: Retrieve several databases through the above search terms, and then import potentially relevant literature into Endnote X8. Researchers will use Endnote X8 to check duplicates and eliminate duplicate literature. Roughly browse documents to prevent careless omissions of the software.
2. The 2nd time of screening the literature: Two researchers will browse titles and abstracts based on inclusion and exclusion criteria, so as to exclude documents that are not related to the research, such as case reports, animal experiments, theoretical researches, studies on non- hepatocirrhosis with splenomegaly. The details of selection process will be shown in the PRISMA flow chart in Figure 1.
3. The 3rd time of screening the literature: Carefully reading the remaining documents and strictly filtering out unqualified documents such as general controlled trials, lacking control group, deficiency of random allocation, incompatible outcome indicator, the appearance of similar data, etc.

As for the literature that cannot be ensured, it would be confirmed by the discussion of the 2 researchers. If they encounter disagreement, the third researcher will resolve it through consultation or questioning. For documents where full text is not available, contact the corresponding author for full text. Read the full-text to determine whether to include it.

#### 2.6.2 Data extraction and management

First of all, a unified data extraction form will be developed. The data extraction of the literature is also independently completed by2 researchers. This process can be completed with the help of excel software. The data extraction form is roughly as follows:

1. Basic information: article title, all authors, publication time, contact information. Article number uses first authors last name plus abbreviation of first name plus year of publication time, for example, related papers published by Carol Ada in August 2019 will be recorded as Ada C. 2019.
2. Research methods: diagnostic criteria, efficacy evaluation criteria, test type, sample size, generation of random number series, implementation of blind method, allocation concealment.
3. Participants: patients age, gender, course of disease, location, number of cases in the control and experimental group.
4. Intervention method: TCM, TCM point, period of treatment, treatment frequency.
5. Outcome measures: primary observation indicators, second-ary observation indicators, improvement of indicators before and after treatment, detailed records of adverse reactions.
6. Source of funding and medical ethics review.
7. Others: number and reasons of dropouts or lost follow-up during the trial, etc.

#### 2.6.3 Risk of bias evaluation

As for the risk of bias in the literature, 2 researchers will independently use the tool for assessing risk of bias recommended by Cochrane Handbook for Systematic Reviews of Interventions 5.1.0 (Cochrane Handbook 5.1.0—Part 2: 8.5–8.7) to assess the quality of the included literature and risk of bias. Evaluation content includes: selection bias (random sequence generation, and allocation concealment), performance bias (blinding of participants and personnel), detection bias (blinding of outcome assessment), attrition bias (incomplete outcome data), reporting bias (selective outcome reporting), and other bias (other sources of bias). Evaluators judge the risk level by carefully reading the full text, which is divided into low risk, high risk, and unknown risk. If the research reported in the literature is not detailed enough, the judgement is usually “unknown risk” of bias. For example, the study uses random number table for grouping, so the random sequence generation will be expressed as “low risk.” If there are any differences, we would consult the third reviewer for solution.

#### 2.6.4 Statistical analysis

The meta-analysis in this review will use RevMan 5.3 and Stata 13.0 software. For the outcome index of the 2 categorical variables, relative risk (Relative risk, RR) will be adopted, and for the outcome index of continuous variables, the mean difference (MD) or standardized mean difference (SMD) will be adopted will a confidence interval (CI) of 95%. Heterogeneity tests will be used for the included studies which will be tested by Chi-Squared test. If *P* ≥ .10 and *I*^2^ ≤ 50%, there is no significant statistical heterogeneity or no statistical difference in heterogeneity, a fixed effect model will be adopted. If *P* < 10 and/or *I*^2^ > 50%, there is significant heterogeneity between studies, a random effect model will be adopted. Further analysis of the source of heterogeneity, if necessary, perform subgroup analysis. There are clinical and methodological differences in the experimental studies. Therefore, random effects models will be selected in this study. Finally, a funnel chart will be drawn to evaluate the publication bias of the literature.

#### 2.6.5 Publication bias

If a result of a meta-analysis contains more than 10 articles and above, we will use a funnel plot to test whether there is a publication bias. If the number of articles included in the study is <10, the publication bias is not significant.

#### 2.6.6 Quality of evidence

The quality of evidence will be assessed by Grades of Recommendations Assessment, Development and Evaluation (GRADE). The evaluation included: downgrade quality of evidence (risk of bias; inconsistency; indirectness; imprecision; publication bias) and upgrade quality of evidence (large effect; plausible confounding; dose–response gradient). The quality of evidence will be divided into 4 levels: high, moderate, low, and very low. Finally, refine the data and use software to edit, analyze, and draw summary of findings table.

## 3 Discussion

In recent years, the number of clinical RCT on splenomegaly due to portal hypertension in cirrhosis have been increasing, however, it is still unsatisfactory in the diagnosis and therapy of the disease. Clinicians have not yet reached a consensus on the treatment principles and evaluation of this disease, and there is a lack of unified standardized standards.^[16]^ At present, there is no large-scale epidemiological investigation on this disease, and there are few reports in related literatures. TCM has rich clinical experience in the treatment of cirrhosis with splenomegaly and has safe and effective curative effect.^[17]^ TCM has a long history and has gone through thousands of years of trials. It has the characteristics of effective and safe.^[18,19]^ It has been used for a long time to treat liver diseases. This therapy mainly stimulates the body's vital qi and harmonizes the Yin, Yang, qi and blood to achieve therapeutic effects. Although the specific mechanism of TCM treatment of hepatic cirrhosis with splenomegaly is still unclear, clinical studies have shown that TCM treatment of hepatic cirrhosis with splenomegaly can significantly improve liver function and reduce portal hypertension. To our knowledge, there has been no comparison of the efficacy of Traditional Chinese medicine in the treatment of liver cirrhosis and splenomegaly. Therefore, we will use a systematic review and meta-analysis to evaluate the efficacy and safety of TCM in the treatment of cirrhosis with splenomegaly.^[20]^ The results of this study can provide a possible evidence-based basis for the TCM treatment of cirrhosis with splenomegaly. In addition, the quality of evidence for the main results will be assessed using a scoring method. We hope that these results can provide clinicians with the basis for TCM treatment of cirrhosis and splenomegaly, and provide the best choice for the treatment of patients.

## Author contributions

**Conceptualization:** Zhaodi Wang, Zhaoxing Chen, Yong Jiang.

**Data curation:** Zhaodi Wang.

**Formal analysis:** Zhaodi Wang, Zhipeng Fan.

**Methodology:** Zhaodi Wang, Zhaoxing Chen.

**Project administration:** Zhaodi Wang.

**Supervision:** Zhaodi Wang, Zhipeng Fan.

**Validation:** Zhaodi Wang, Yong Jiang.

**Writing – original draft:** Zhaodi Wang, Yong Jiang.
